# Beyond ritual: gender visibility and the commemoration of Jane Austen across cultural contexts

**DOI:** 10.3389/fsoc.2026.1941056

**Published:** 2026-08-13

**Authors:** Dilbar Khaydarova

**Affiliations:** Department of English Literature and Translation Studies, Faculty of Foreign Languages, Bukhara State University, Bukhara, Uzbekistan

**Keywords:** feminist memory, gender visibility, institutional commemoration, International Women's day, Jane Austen, literary heritage

## Abstract

This article contends that commemorations of women's achievements should not be dichotomized as either genuine empowerment or mere ritual. Using the commemoration of Jane Austen as a case study, gender visibility is conceptualized as an ongoing institutional process rather than a singular event. Three institutional tracks are identified: heritage sites, hybrid fan-scholar communities, and academic institutions, spanning the UK, US, and Uzbekistan. Drawing on Durkheim's and Collins's sociology of ritual, as well as feminist memory scholarship, the analysis demonstrates that symbolic recognition and structural change are differentiated outcomes shaped by institutional form rather than competing judgments of a single event. The article further explores the implications of this framework for tokenism, intersectionality, and canon change.

## Introduction

Observances such as International Women's day are frequently characterized as either empowering or merely symbolic, yet this debate typically focuses on single day events and seldom addresses visibility constructed through sustained institutional engagement. This article extends the discussion to heritage sites, literary societies, and university curricula organized around a single writer, thereby illustrating how institutional commemoration operates over time.

The central claim is that empowerment and ritual are not opposites: repetition is precisely what lets each new generation rediscover and reinterpret a woman writer, so ritual often functions as a delivery mechanism for empowerment rather than its negation. Austen is a useful test case because her commemoration runs along three separate tracks heritage bodies preserving her physical legacy, hybrid societies blending scholarship with fan culture, and university departments, including in non-Anglophone settings such as Uzbekistan, sustaining her through teaching and research.

Writing from a Central Asian institutional context where Austen enters through post-Soviet curricular structures rather than heritage tourism or fan culture offers a different perspective largely absent from existing commemoration scholarship, which has concentrated on Anglo-American settings. This position informs the comparative framework developed below. The analysis draws on publicly available institutional sources: museum event programming, society records, conference themes, and curricular material rather than on a systematic sample; the three cases are treated as illustrative of institutional variation rather than as a representative cross-section, a limitation addressed further in the Discussion.

## Theoretical framework

Durkheim (1995) account of ritual as the mechanism by which a group periodically reaffirms its shared symbols, and Collins (2004) argument that repeated interaction rituals generate and redistribute emotional energy around such symbols, together suggest that commemorative repetition is constitutive rather than incidental. On this view, repetition is not evidence that recognition has stalled at the symbolic level; it is the mechanism by which a symbol—here, Austen as a woman writer—continues to accumulate meaning across successive cohorts of participants. Feminist memory scholarship complicates this picture by insisting that whose memory work counts, and which institutions are authorized to perform it are themselves gendered (Hirsch and Smith, 2002). Read together, these literatures imply that symbolic recognition and structural change are not competing outcomes of a single commemorative act, but differentiated products of the institutional form that repetition takes. In this framework, institution type is the key variable that shapes whether commemoration remains symbolic or becomes structurally embedded. Institutional form refers to the structural conditions under which repetition happens: is participation voluntary or required, is engagement tied to a calendar or ongoing, does it reach a self-selecting public or a mandated one. It is these conditions, not repetition on its own, that decide whether symbolic recognition turns into structural change.

## Literature review

Scholarship on Austen's posthumous reception and fan culture (Johnson, 2012; Yaffe, 2013) and heritage or memory studies more broadly have developed largely in parallel, each treating Austen commemoration as either a literary-historical curiosity or a heritage-tourism case rather than as a single object comparable across institutional types. Johnson (2012) traces how Austen's reputation shifted from a modest nineteenth-century readership to a twentieth century “cult,” but does so primarily within Anglo-American literary and heritage contexts; Yaffe (2013) documents the contemporary fan community in similar geographic terms. This mirrors a broader disciplinary silo between literary attention to women writers and the sociological study of gendered commemoration (Hirsch and Smith, 2002). Comparative work that follows one figure across multiple institutional pathways—heritage, fandom, and academe—is rarer still. This article addresses that gap by tracing Austen's commemoration across three national contexts, drawing on museum publications, society records, conference programming, and university curricula.

## Analysis

The three pathways discussed below illustrate variation within one author's reception. They are not offered as a representative sample of institutional types for women writers in general. The goal is to test a comparative framework, not to generalize beyond this case.

### Heritage-based

Jane Austen's House, Chawton House, and the Jane Austen Festival generate strong symbolic visibility through preservation and events, but the heritage track shows a more layered pattern than pure repetition-as-stasis. The festival's public programming promenades, balls, costume workshops has remained largely stable since 2001, oriented toward tourism and pageantry rather than reinterpretation, consistent with the fan-pilgrimage dynamic Johnson (2012) associates with earlier phases of Austen's heritage reception. Yet a parallel institutional practice tells a different story: Jane Austen's House has built a recurring, cross-institutional March 8th tradition, partnering annually with the Brontë Parsonage Museum, Chawton House, and Elizabeth Gaskell's House to reframe Austen within a rotating set of feminist and literary-historical themes—women's private correspondence in 1 year, adaptation and legacy in another. The heritage track, then, contains both functions the literature associates separately with ritual: the festival exemplifies repetition as stable commemoration, while the museum's IWD programming exemplifies repetition as a vehicle for reinterpretation. This internal split suggests that heritage institutions are not monolithically conservative; the mechanism depends on which sub-institution, and which calendared occasion are doing the repeating (Jane Austen's House, 2022, 2023, 2024).

### Hybrid

JASNA, the Jane Austen Society of North America, merges fan enthusiasm with scholarly infrastructure, turning informal engagement into durable outputs such as conferences and publications. JASNA's own conference themes evidence repetition functioning as reinterpretation rather than mere reiteration: the 2017 AGM, marking Austen's bicentenary, explicitly framed her legacy through generational reinterpretation, linking her reception history to social movements including the suffragettes and feminist scholarship, while other cycles 2018's focus on constancy and hope, 2022's domestic themes in Sense and Sensibility centered more narrowly on individual novels. The same recurring institution, across repeated cycles, has produced markedly different readings of the same author (Jane Austen Society of North America (JASNA), 2017, 2025; JASNA Southwest Region, n.d.). The 2025 anniversary—the Baltimore AGM and Bath's expanded festival edition shows how both hybrid and heritage tracks absorbed the same milestone differently.

### Academic-based

Austen is embedded in Uzbek university curricula (world literature courses, text-analysis seminars at Bukhara State University) as a socio-economic and feminist text, not just domestic fiction—a form of structural embedding shaped by local post-Soviet, Central Asian conditions of language and institutional access, and distinct in character from her UK/US reception. This distinctiveness is a matter of mechanism, not depth. United Kingdom and United States tracks embed Austen through voluntary institutions that people opt into, such as heritage bodies and fan societies. The Uzbek case embeds her through compulsory curricular structures instead. These reach students regardless of whether they came in with any interest in Austen or in feminist literary criticism. A brief parallel is instructive here: Uzbekistan has its own tradition of honoring the poet Zulfiya through a state prize awarded on March 8th, recognizing women's literary and scholarly achievement through national cultural policy rather than through heritage tourism or fan culture (Government of Uzbekistan, 2026). Read alongside the Austen case, this suggests that state-curricular and prize-based institutionalization of women's literary recognition operates on a different logic top-down and compulsory than the voluntary, self-selecting heritage and fan institutions that dominate the UK/US literature, a distinction future comparative work could develop further.

Across all three pathways, the analysis flags a tokenism risk: Austen often serves as the default “representative” woman writer, which may substitute for broader canon change rather than demonstrate that it is happening.

## Discussion

The empowerment-vs.-ritual binary assumes a single event to be judged once; institutional commemoration instead produces a spread of outcomes—some purely symbolic, some structural, most a mix of the two. The proposed three-pathway framework offers a reusable tool for assessing this elsewhere, and it refines the guiding question: does a given institutional pathway widen recognition for women writers broadly, or does it simply supply one convenient example and stop there?

Two limitations bound these claims. First, the case selection is illustrative rather than systematic: a single author's commemoration across three national contexts cannot establish how representative these patterns are of women's literary commemoration more broadly. Second, the analysis relies on institutional self-representation—event pages, programming records, curricular descriptions—rather than on audience reception data, so claims about what each pathway accomplishes for tokenism or canon change remain inferential.

### Future directions

The three-pathway framework proposed here—heritage, hybrid, and academic—is intended as a comparative tool rather than a closed taxonomy, and its value will depend on being tested against cases beyond Austen. Three extensions seem quite promising. First, systematic comparison across multiple women writers commemorated through similar institutional forms would show whether the heritage/hybrid/academic split holds generally or is an artifact of Austen's particular reception history. Second, reception research could test something this article cannot test on its own: interviews or survey work with the publics each pathway reaches. Does the reinterpretation credited to certain institutional practices, like the museums' rotating IWD themes or JASNA's shifting conference foci, actually register with participants? Or does it only show up at the level of institutional programming? Third, the Uzbek case invites further comparative work on state-sponsored, curricular, and prize-based mechanisms of women's literary recognition in non-Western and post-Soviet contexts, where the voluntary heritage-and-fandom model that dominates Anglo-American commemoration scholarship does not straightforwardly apply. Pursuing these would help establish whether the tokenism risk identified here is a general property of single-author commemoration or is instead mitigated by institutional arrangements—a question this article raises but cannot, on its own evidence, resolve.

## Conclusion

Jane Austen's lasting visibility shows that remembering women writers depends on institutional structures, not just commemorative moments and the empowerment-or-ritual question is better answered institution by institution than as a blanket judgment.

## Data Availability

The original contributions presented in the study are included in the article/supplementary material, further inquiries can be directed to the corresponding author.
